# The association between serine hydroxymethyl transferase 1 gene hypermethylation and ischemic stroke

**DOI:** 10.17305/bjbms.2020.5188

**Published:** 2021-08

**Authors:** Junnan Wang, Junqing Gu, Yi Huang, Yuanjian Fang, Jinghui Lin

**Affiliations:** 1 Department of Cardiology, Yuyao People’s Hospital, Ningbo, Zhejiang, China; 2 Department of Internal Medicine, Yuyao People’s Hospital, Ningbo, Zhejiang, China; 3 Department of Neurosurgery, Ningbo First Hospital, Ningbo, Zhejiang, China; 4 School of Medicine, Zhejiang University, Hangzhou, Zhejiang, China

**Keywords:** Ischemic stroke, DNA methylation, serine hydroxymethyl transferase 1, homocysteine, high-density lipoprotein, sex

## Abstract

This study aimed to determine the correlation between serine hydroxymethyl transferase 1 (*SHMT1*) gene methylation and ischemic stroke. A total of 202 age- and sex-matched individuals were included. Quantitative methylation-specific polymerase chain reaction (qMSP-PCR) was used to analyze the DNA methylation level. The plasma homocysteine (Hcy) concentration was much higher in ischemic cases than in controls (*p* = 0.009), while the high-density lipoprotein (HDL) levels in stroke cases were considerably lower than in controls (*p* = 0.005). A significantly higher level of *SHMT1* methylation was observed in the ischemic strokes (58.82 ± 17.83%) compared to that in the controls (42.59 ± 20.76%, *p* < 0.001). The *SHMT1* methylation level was strongly correlated with HDL concentration in the healthy controls (r = 0.517, *p* < 0.001), while the high plasma level of Hcy showed strong association with *SHMT1* methylation in ischemic strokes (r = 0.346, *p* < 0.001). Receiver operating characteristic (ROC) analysis of curve indicated that *SHMT1* methylation has been an acceptable indicator for ischemic stroke in female patients [all sexes, area under the curve (AUC) = 0.71, *p* < 0.001; male patients AUC = 0.62, *p* = 0.032; and female patients AUC = 0.79, *p* < 0.001] and in all ages (AUC = 0.71, *p* < 0.001). In our samples, DNA methylation levels of the *STHMI* gene were significantly correlated with ischemic stroke in Han Chinese. *STHMI* hypermethylation was significantly associated with the high Hcy concentration in ischemic stroke and had value as a potential indicator for female ischemic stroke.

## INTRODUCTION

Ischemic stroke is an acute cerebrovascular disease associated with extremely high mortality. It has become the leading cause of disease-related death in China [1]. However, the pathogenesis of ischemic stroke is not fully understood. Ageing, hypertension, hyperlipidemia [2], genetics [3], and lifestyle [4] are reported as the main risk factors for ischemic stroke [5]. Several studies have shown that genetic and epigenetic factors play key roles in the development of ischemic stroke. DNA methylation was previously suggested to be involved in stroke pathogenesis by affecting the expression of stroke-related genes [6]. For example, one study showed that the low level of long interspersed nucleotide element 1 gene methylation is associated with a high risk for ischemic stroke in men [7], while another suggested that matrix metalloproteinase-2 gene demethylation is associated with ischemic stroke in a sex and stroke subtype-specific manner [8].

The serine hydroxymethyl transferase 1 (*SHMT1*) gene plays an important role in folic acid metabolism. *SHMT1* can promote the formation of 5-methyltetrahydrofolate during folate metabolism and the homocysteine (Hcy) metabolism pathway in the methionine cycle [9]. Hcy was shown to increase brain lesions after stroke [10] and has been suggested as a high risk factor for the onset of stroke [11]. *SHMT1* variants may be involved in Hcy metabolism and, therefore, contribute to an increased risk of ischemic stroke [12]. The methylation of *SHMT1* gene may affect the expression of SHMT1 [13], a protein that plays a key role in promoting the conversion of serine and tetrahydrofolate to glycine and 5,10-methylenetetrahydrofolate [14]. Moreover, SHMT1 expression was shown to be associated with phosphate-induced vascular smooth muscle cell calcification [15] and a *SHMT1* variant was suggested as a risk factor in early-onset ischemic stroke [16]. In addition, the altered expression of SHMT1 could block folic acid metabolism and abnormal Hcy remethylation pathways. This causes excessive accumulation of Hcy, which could increase the risk of ischemic stroke [17]. A previous study showed that *SHMT1* promoter hypermethylation was confirmed in both, patients with essential hypertension, and patients with hyperhomocysteinemia [13]. However, DNA methylation of this gene had not been examined in the context of ischemic stroke. Therefore, we hypothesized that DNA methylation of *SHMT1* would be associated with ischemic stroke case–control status. The purpose of this study was to determine the correlation between *SHMT1* gene methylation and ischemic stroke in a Chinese population. We also identified the effects of *SHMT1* methylation on Hcy and circulating lipids in ischemic stroke patients.

## MATERIALS AND METHODS

### Study participants

The study was approved by the Ethics Committee of the Ningbo First Hospital (2014-002 and 2017-R028) and written informed consent was given by all participants. The study group included 101 patients with ischemic stroke (51 males and 50 females, mean age 61.07 ± 11.56 years) and 101 age- and sex- matched healthy volunteers (51 males and 50 females, mean age 62.49± 8.93 years). All the individuals were recruited from the stroke center of Ningbo First Hospital between September 2013 and December 2019. The diagnoses of ischemic stroke patients were confirmed based on international standardized definitions, and magnetic resonance imaging (MRI) and cranial computed tomography (CT) scan findings. The control group individuals were recruited from the health center and those with serious liver disease, kidney disease, or any cerebral vascular diseases were excluded from the study.

### Biochemical measurements

General information for the individuals, including sex, age, hypertension (self-reported a history of antihypertensive drug use), diabetes (self-reported positive history of type 1 or type 2), drinking history (self-reported positive history of drinking, more than 50 ml drinks/week), smoking history (self-reported positive history of smoking), and body mass index (BMI), were obtained. A total of 5 mL fasting venous blood was collected from the volunteers on the first morning of cerebral infarction and used for the detection of biochemical indicators; the remaining blood was used for DNA extraction. The plasma levels of Hcy were measured by cycling enzymatic method; triglycerides (TGs), total cholesterol (TC), high-density lipoprotein (HDL), and low-density lipoprotein (LDL) were determined by the enzymatic method; and apolipoprotein A (ApoA), apolipoprotein B (ApoB), and apolipoprotein E (ApoE) were estimated through the transmission turbidimetric method. All the biochemical measurements were tested using an automatic biochemical analyzer (AU2700; Olympus, Japan).

### DNA methylation data collection

Blood DNA was extracted using magnetic bead isolation method and performed on the Lab-Aid 820 Nucleic Acid Extractor (Xiamen Zhishan Biological Technology Co., Ltd., China). DNA methylation was measured using quantitative methylation-specific polymerase chain reaction (qMSP-PCR) assay and performed on the LightCycler 480 (Roche Diagnostics, Mannheim, Germany). *ACTB* was selected as the internal reference gene to standardize the amount of target DNA, and completely methylated DNA was used as a positive control [18]. A fragment (GRCh37/hg19, chr17: 18266824–18266911) located in the *SHMT1* CpG island was selected to determine the level of DNA methylation. The qMSP primers and PCR reaction conditions for *SHMT1* (forward primer: 5’-cgagtttaggaaggtgtatt-3’, reverse primer: 5’-ccatacttaactacgctctc-3’) and *ACTB* (forward primer: 5’-ccagtttaggaaggtgtatt-3’, reverse primer: 5’-ccatacttaactacgctctc-3’) were the same as those presented in a previous study [13]. The percentage of methylated reference (PMR) was calculated by the 2^−ΔΔCt^ method to represent gene methylation (methylation percentage ranged from 0% to 100%). [19], in which ΔΔCt = sample DNA (Ct_target gene_ – Ct _ACTB_) − fully methylated DNA (Ct _target gene_ – Ct _ACTB_) [20].

### Statistical analysis

The data were expressed as mean ± standard deviation (SD) or number and analyzed by the t-test or Pearson Chi-square. The association between *SHMT1* methylation and clinical data was assessed using the Spearman’s (Hcy) or Pearson’s (other factors normally distributed) correlation test and multivariate binary logistic regression analysis after adjustment for age, sex, BMI, Hcy, TG, TC, HDL, and LDL. In stratified analysis, ischemic stroke potential modifiers, such as sex (male or female) and age (<60 or ≥60 years), were assessed. The receiver operating characteristic (ROC) curve test was used to evaluate the sensitivity of *SHMT1* methylation for ischemic stroke diagnosis. Data were analyzed using SPSS V20.0 (Armonk, NY, USA) and figures plotted using GraphPad Prism V8.0 (La Jolla, CA, USA). Statistical significance was considered at *p* < 0.05.

## RESULTS

The clinical characteristics of all participants are shown in Table 1. The BMI was significantly different between the two groups (cases vs. controls: 24.45 ± 3.11 kg/m^2^ vs. 23.43 ± 3.10 kg/m^2^, respectively, *p* = 0.020). The plasma Hcy concentration was much higher in ischemic cases (18.48 ± 10.29 μmol/L) than in controls (15.27 ± 6.35 μmol/L, *p* = 0.009), while the HDL levels in stroke cases (1.05 ± 0.31 mmol/L) were considerably lower than in controls (1.16 ± 0.24 mmol/L, *p* = 0.005). In contrast, LDL concentration was higher in ischemic cases (2.91 ± 0.78 mmol/L) than in controls (2.61 ± 0.84 mmol/L, *p* = 0.011).

**TABLE 1:**
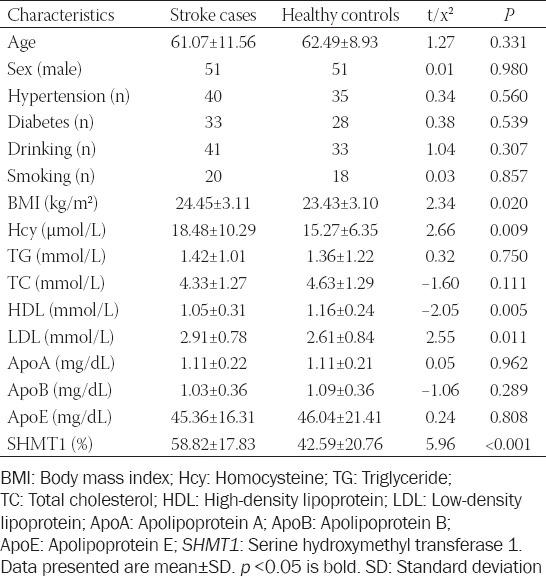
The clinical characteristics for all participants

**FIGURE 1:**
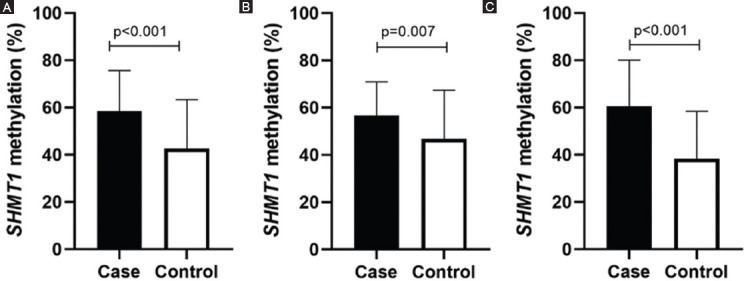
Comparison of serine hydroxymethyl transferase 1 methylation (*SHMT1*) levels between cases and controls in male and female. (A) All groups, ischemic stroke versus controls: 58.82 ± 17.83 versus 42.59 ± 20.76, *p* < 0.001. (B) Male groups, ischemic stroke versus controls: 56.59 ± 14.35 versus 46.82 ± 20.67, *p* = 0.007. (C) Female groups, ischemic stroke versus controls: 60.59 ± 19.51 versus 38.28 ± 20.16, *p* < 0.001.

**FIGURE 2:**
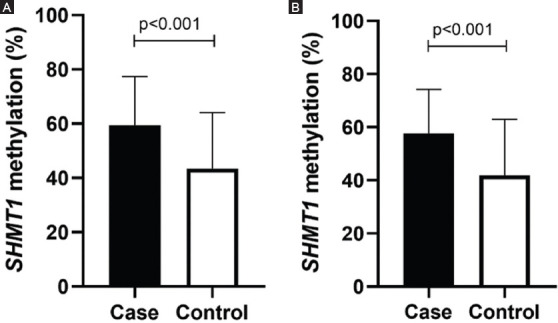
Comparison of serine hydroxymethyl transferase 1 methylation (SHMT1) level in different ages. (A) Age ≤ 60 years, ischemic stroke versus controls: 59.46 ± 17.94 versus 43.49 ± 20.63, p < 0.001. (B) Age > 60 years, ischemic stroke versus controls: 57.73 ± 16.47 versus 41.90 ± 21.02, p < 0.001.

As shown in Figure 1, the *SHMT1* methylation levels were significantly higher in the stroke patients (58.82 ± 17.83%) than in the controls (42.59 ± 20.76%, *p* < 0.001). Subgroup analysis by sex demonstrated that *SHMT1* methylation levels in both male (56.59 ± 14.35%) and female (60.59 ± 19.51%) cases were higher than those in controls (46.82 ± 20.67%, *p* = 0.007 and 38.28 ± 20.16%, *p* < 0.001, respectively). Similarly, subgroup analysis by age indicated significantly higher levels of *SHMT1* methylation in ischemic stroke patients than in controls in both the evaluated age groups (age ≤60 years, ischemic stroke vs. controls: 59.46 ± 17.94% vs. 43.49 ± 20.63%, *p* < 0.001; age > 60 years, ischemic stroke vs. controls: 57.73 ± 16.47% vs. 41.90 ± 21.02%, *p* < 0.001, Figure 2).

**FIGURE 3:**
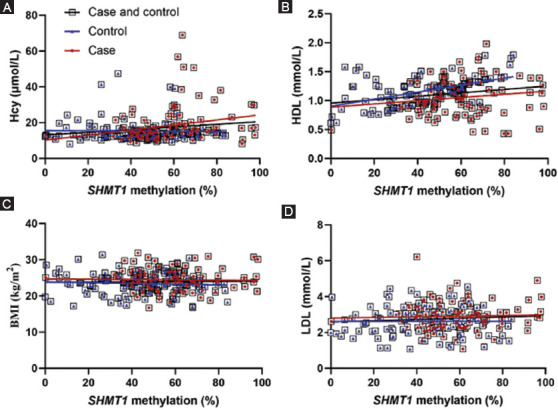
The relationships between serine hydroxymethyl transferase 1 (*SHMT1*) methylation and homocysteine (Hcy), high-density lipoprotein (HDL), BMI, and low-density lipoprotein (LDL) in different groups. (A, B) *SHMT1* methylation was associated with Hcy in stroke cases (stroke cases, *p*= 0.034, controls, *p* = 0.950; total, *p* = 0.011, (A) and HDL in controls (stroke cases, *p* = 0.146, controls, *p* < 0.0001, total, *p* = 0.001, (B) using regression analysis. (C, D) BMI (stroke cases, *p*= 0.821, controls, *p* = 0.937; total, *p* = 0.673, (C) and LDL (stroke cases, *p* = 0.603, controls, *p* = 0.646; total, *p* = 0.221, (D) showed no association with *SHMT1* methylation though regression analysis.

Next, we explored the relationship between *SHMT1* methylation and biochemical indicators. As shown in Table 2, significant correlations were found between *SHMT1* methylation and HDL concentration in controls (r = 0.517, *p* < 0.001), but not in ischemic cases (r = 0.153, *p* = 0.127, Pearson’s test). In contrast, the plasma level of Hcy showed strong association with *SHMT1* methylation in ischemic stroke patients (r = 0.346, *p* < 0.001), but not in controls (r = 0.037, *p* = 0.715, Spearman’s test). In multivariate analysis, the levels of *SHMT1* methylation (*p* < 0.001, OR [95% CI] = 1.07 [1.04-1.09]) and HDL (*p* < 0.001, OR [95% CI] = 0.02 [0.004-0.13], Table 3) were strongly related to ischemic stroke. The distributions of BMI, Hcy, HDL, and LDL are shown in Figure 3. *SHMT1* methylation was also associated with Hcy in stroke cases (stroke cases, *p* = 0.034, controls, *p* = 0.950; total, *p* = 0.011) and HDL in controls (stroke cases, *p* = 0.146, controls, *p* < 0.0001, total, *p* = 0.001) using regression analysis adjusted for age, sex, BMI, Hcy, HDL, and LDL. BMI (stroke cases, *p* = 0.821, controls, *p* = 0.937; total, *p* = 0.673) and LDL (stroke cases, *p* = 0.603, controls, *p* = 0.646; total, *p* = 0.221) showed no association with *SHMT1* methylation though regression analysis.

**TABLE 2:**
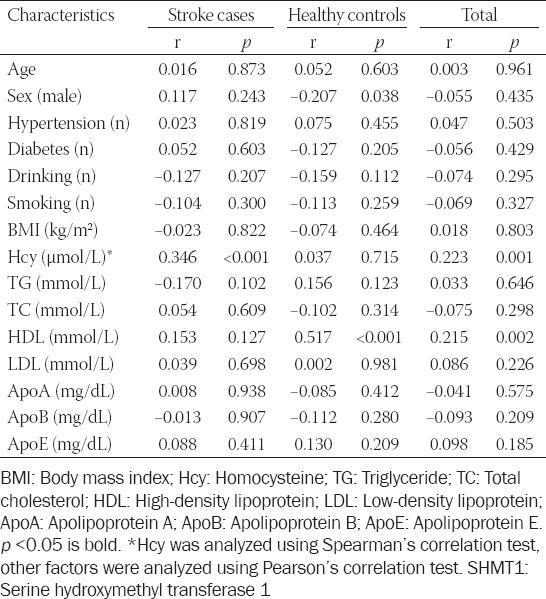
Correlation analysis between *SHMT1* methylation and relative factors

**TABLE 3:**
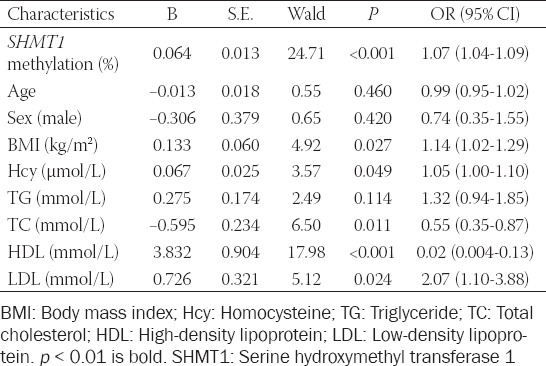
Multivariable logistic regression analysis of association between *SHMT1* methylation and the risk of ischemic stroke.

**FIGURE 4:**
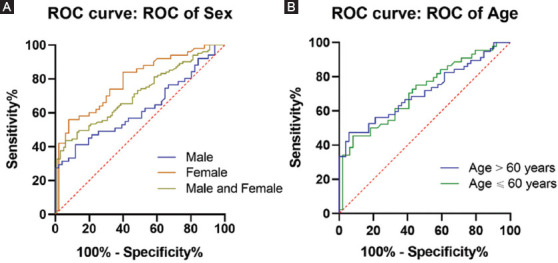
ROC curves of serine hydroxymethyl transferase 1 gene DNA methylation in ischemic strokes. ROC: Receiver operating characteristic, AUC, area under the curve. (A) In the male and female patients, AUC = 0.71, *p* < 0.001; male patients, AUC = 0.62, *p* = 0.032; and female patients, AUC = 0.79, *p* < 0.001. (B) In the group of age ≤ 60 years, AUC = 0.71, *p* < 0.001; age > 60 years, AUC = 0.71, *p* < 0.001.

As shown in Figure 4, the ROC analysis of curve showed that *SHMT1* methylation had acceptable diagnostic value for ischemic stroke regardless of sex (area under the curve [AUC] = 0.71, *p* < 0.001; male patients, AUC = 0.62, *p* = 0.032; and female patients, AUC = 0.79, *p* < 0.001). The age subgroup analysis suggested similar results in all ages (age ≤ 60 years, AUC = 0.71, *p* < 0.001; age > 60 years, AUC = 0.71, *p* < 0.001).

## DISCUSSION

In the present study, we explored the association between *SHMT1* methylation and ischemic stroke. Our results showed that the methylation levels of *SHMT1* were much higher in ischemic stroke patients than in healthy controls. *STHMI* hypermethylation was significantly associated with plasma Hcy concentration in ischemic stroke patients. In addition, ROC analysis suggested that *SHMT1* methylation may be a useful predictor for female ischemic stroke.

Hcy was a sulfur-containing amino acid produced by the demethylation of methionine [21]. The previous studies had demonstrated that plasma Hcy was an independent risk factor for stroke [22,23]. Hcy could increase the risk of developing ischemic stroke and lacunar infarction [23]. Plasma Hcy concentration in the acute phase was suggested to associate with ischemic stroke mortality in patients [24]. *SHMT1* was an important supplier of carbon unit in the process of folate metabolism [25]. The *SHMT1* hypermethylation could reduce the expression of SHMT1 [13] causing folic acid metabolism and Hcy remethylation pathways to be blocked [26]. Subsequently, excessive accumulation of Hcy caused to hyperhomocysteinemia [27], which led to stroke. Our results showed that the plasma Hcy concentration was much higher in ischemic stroke patients and was significantly associated with *SHMT1* methylation. These associations were consistent with the above inference.

Genetic factors and environmental factors are known to participate in the pathological process of ischemic stroke development, with several studies suggesting that patients with a high BMI may have a higher risk for ischemic stroke [28] due to higher blood lipid levels [29]. In fact, serum levels of HDL and LDL are significantly associated with increased risk of ischemic stroke [30] and people with lower concentration of LDL have a lower risk of subsequent stroke [31]. The current study showed that HDL concentration was associated with the ischemic stroke severity, and the level of HDL was much higher in severe stroke compared with mild stroke [32]. HDL has been suggested as a new target for stroke treatment, which might impact the care of stroke patients [33]. Other studies showed that the high level of HDL cholesterol was showed to associate with a decreased risk of ischemic stroke [34]. Low level of HDL cholesterol was associated with increased risk of ischemic stroke [35]. In this study, the results showed that BMI and LDL levels were significantly higher in ischemic stroke patients. Moreover, the HDL concentration was lower in the ischemic stroke patients and the *SHMT1* methylation levels were associated with HDL concentration in controls. The previous studies showed that altered Hcy metabolism can have an effect on HDL levels [36], which may partly explain this association in the controls.

Age and sex are well known risk factors for ischemic stroke [37]. With age, the risk of stroke in the elderly increased significantly, and other conditions related to aging might aggravate clinical and functional consequences [38]. Margaret et al. showed that older age (age >60 years) of stroke onset is associated with greater disability [39]. Stroke has shown to affect females more than males because of their physical characteristics and living habits [40]. Females have also shown to have a much higher prevalence of stroke after age 45 [41], which largely due to a sharp increase risk in older postmenopausal female [42]. The previous studies suggested that there were sex differences in methylation of ischemic stroke-related genes [43]. Such as, the DNA methylation level of long interspersed nucleotide element 1 gene was associated with a higher risk for ischemic stroke in male, but not in female patients [7]. Our results showed that *SHMT1* methylation levels were associated with ischemic stroke in both sexes, regardless of age. Moreover, the ROC analysis showed that *SHMT1* methylation had better diagnostic value in female patients.

There are some limitations to our study that need to be considered. First, this was a candidate gene study, we had not studied how DNA methylation affected gene expression and other factors (such as cell-type heterogeneity), *SHMT1* methylation and expression. These confounding factors should be studied in the future. Second, *SHMT1* played an important role in the folic acid metabolism, so the function of other genes should be considered in future research. Third, the sample size was small and, therefore, we could not find any association between ischemic stroke and other clinical characteristics such as smoking, drinking, hypertension, diabetes, and blood lipids. Thus, future studies with larger samples that include multiple ethnic populations are needed to confirm our findings.

## CONCLUSION

In our samples, DNA methylation levels of the *STHMI* gene were significantly correlated with ischemic stroke. *STHMI* hypermethylation was significantly associated with Hcy concentration in ischemic stroke and was shown to be a potential diagnostic tool for ischemic stroke in females. However, future studies to confirm these findings are required prior to potential clinical application of these results.
